# Summary of the Intercomparison of the Force Standard Machines of the National Institute of Standards and Technology, USA, and the Physikalisch-Technische Bundesanstalt, Germany

**DOI:** 10.6028/jres.096.029

**Published:** 1991

**Authors:** Simone L. Yaniv, A. Sawla, M. Peters

**Affiliations:** National Institute of Standards and Technology, Gaithersburg, MD 20899 USA; Physikalisch-Technische Bundesanstalt, Germany

**Keywords:** deadweight machines, force measurements, force standards, force transducers

## Abstract

A comparison of force measurements performed at the National Institute of Standards and Technology, USA, and at the Physikalisch-Technische Bundesanstalt, Germany is reported. The focus of the study was the intercomparison of the forces realized by the two Institutes rather than the measurement process. The transfer standards used in the comparison consisted of force transducers and associated readout instrumentation. The results of the intercomparison reveal that over a range of 50 kN to 4.5 MN, the forces realized at NIST and at PTB compare favorably. For forces up to 900 kN the agreement is within ±40 ppm; above 900 kN the agreement is within ± 100 ppm.

## 1. Introduction

This paper summarizes the results of a comparison of force measurements performed at the National Institute of Standards and Technology (NIST), USA, and at the Physikalisch-Technische Bundesanstalt (PTB), Germany. A detailed description of the study can be found in Ref. [1].

The objective of the study was to determine the comparability of the forces realized by the two Institutes over a range of 50 kN to 4.5 MN, so that the results of force transducer calibrations performed at one Institute would be more readily accepted by the other. The need for the comparison was acute as there are significant differences in the force machines used by the two Institutes. At NIST the forces applied are generated by deadweights over the entire range included in the study. At PTB forces up to 1 MN are applied by deadweights but higher forces are generated by means of a hydraulic force-multiplication system.

The overall program for the intercomparison was developed jointly by NIST and PTB. The protocol used during the measurements was developed by PTB. Planning for the program began late in 1988. Initial measurements of all the force transducers involved in the comparison were carried out first at PTB in September 1989. These initial measurements were followed by a set of similar measurements at NIST in October 1989. To verify the stability of the force transducers used during the comparison, a final set of measurements was obtained at PTB in November 1989.

## 2. Force Standard Machines

The force standard machines of both Institutes have been described in details in Refs. [1–4]. Accordingly, only a brief description of the machines is given here.

### 2.1 The NIST Deadweight Force Standard Machines

**Figure 1:**
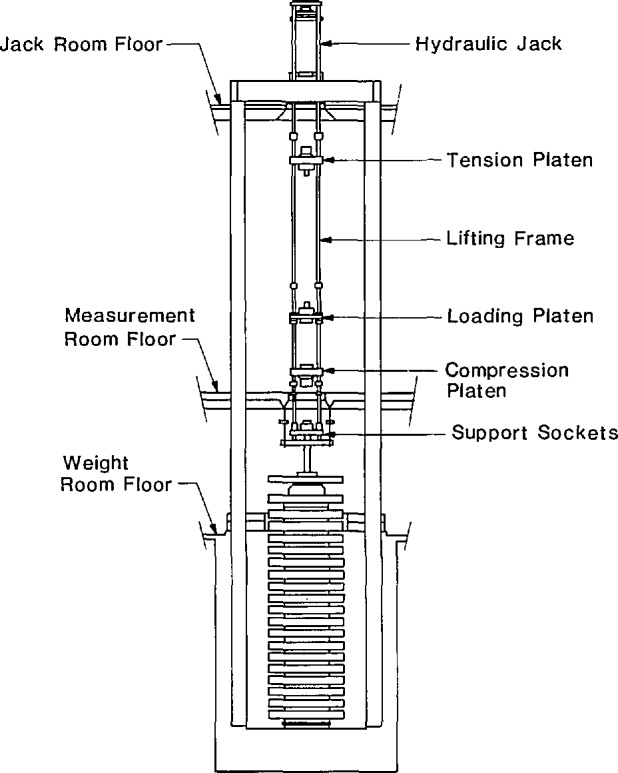
The NIST 1 Mlbf deadweight machine.

**Figure 2:**
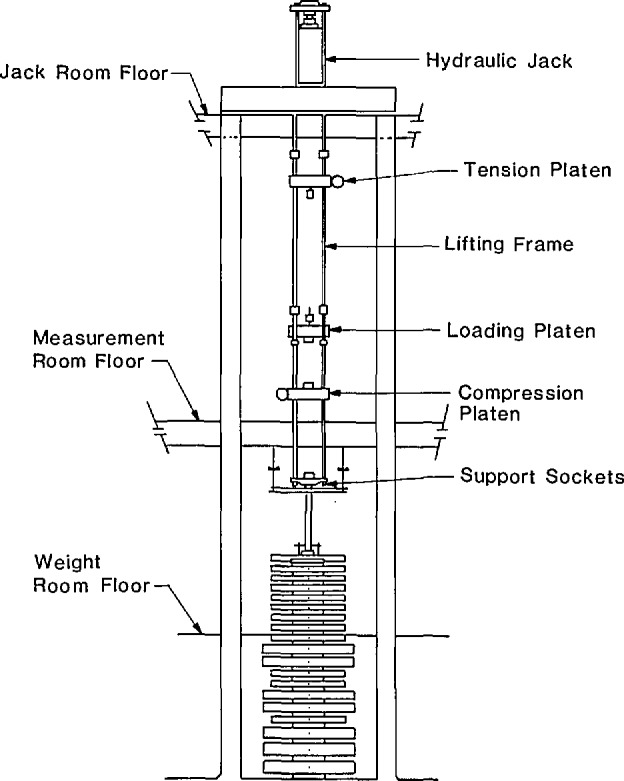
The NIST 300 klbf deadweight machine.

**Figure 3:**
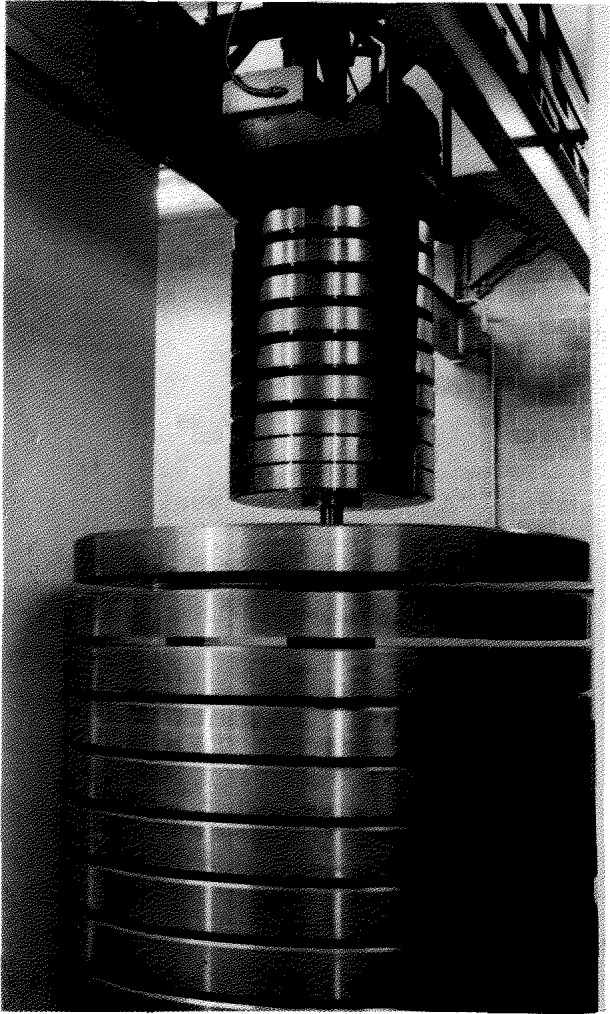
The NIST 112 klbf deadweight machine.

Only the three largest NIST deadweight machines were included in the intercomparison. These machines, known as the 1 Mlbf, the 300 klbf and the 112 klbf machines have been described in Refs. [1,2]. The most important features of these machines are summarized in Table 1. Drawings of the two largest machines are given in Figs. 1 and 2, and a photograph of the 112 klbf machine is given in Fig. 3.

The estimated total uncertainty of the vertical component of force applied by any weight is 0.002 percent. If corrections are made for the actual air density during a calibration and for the actual adjusted mass of each weight, the uncertainty of the applied force can be reduced to 0.001 percent.

**Figure 4:**
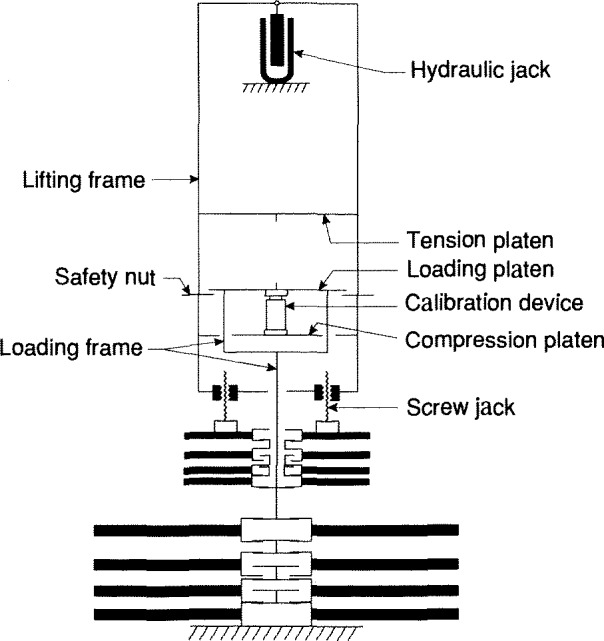
Schematic drawing of the three largest NIST dead-weight machines.

The schematic drawing in Fig. 4 illustrates the operating principles of the deadweight machines. Each of the three machines has a stack of large weights, represented by the lower stack of larger weights in Fig. 4. Only the 112 klbf machine also has a second stack of smaller weights that are operated by screw jacks. To apply a deadweight load, the hydraulic jack raises the lifting frame and the loading frame, acting through the device being calibrated in either compression or tension.

As the loading frame is raised, the large weights are picked up in sequence, beginning with the top weight of the stack. In the 112 klbf machine, the smaller weights are lowered onto the loading frame by the screw jacks, in sequence, beginning with the bottom weight of the stack. The time required to apply the capacity load in each machine is given in Table 1. The vertical positions of the compression and tension platens of the lifting frame are adjusted as required to fit each calibration setup. The maximum setup space in each machine is given in Table 1. The safety nuts are adjusted so that they will support the deadweight load if the calibration setup becomes unstable. The vertical members of the loading frames are not clearly visible in Figs. 1 and 2 because the widths of the loading frames are approximately equal to the widths of the respective lifting frames.

### 2.2 The PTB Force Standard Machines

To cover the force range included in the inter-comparison, two PTB force standard machines were utilized. In the first machine, the 1 MN machine, forces are generated by deadweights. In the second machine, the 15 MN machine, forces are generated by means of a hydraulic force multiplication system. These machines have been described in detail in Refs. [1,3,4].

**Figure 5:**
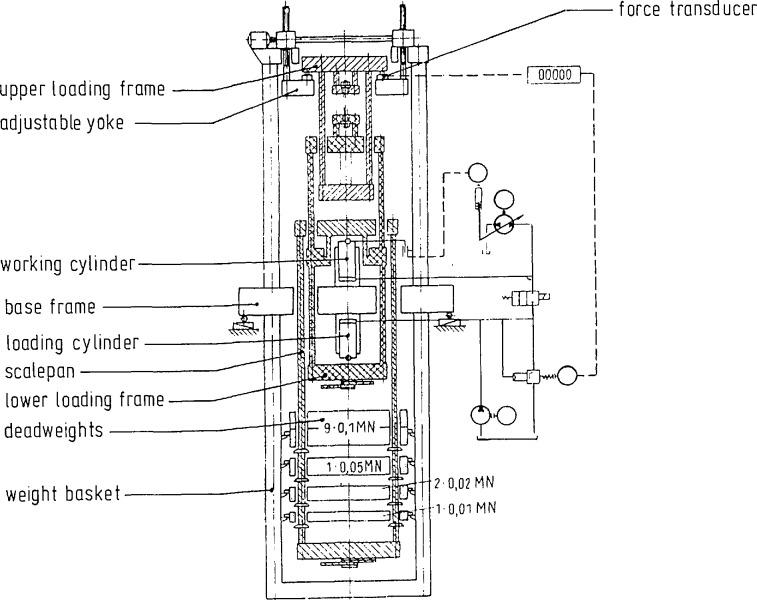
Schematic diagram of the PTB 1MN deadweight machine.

**Figure 6:**
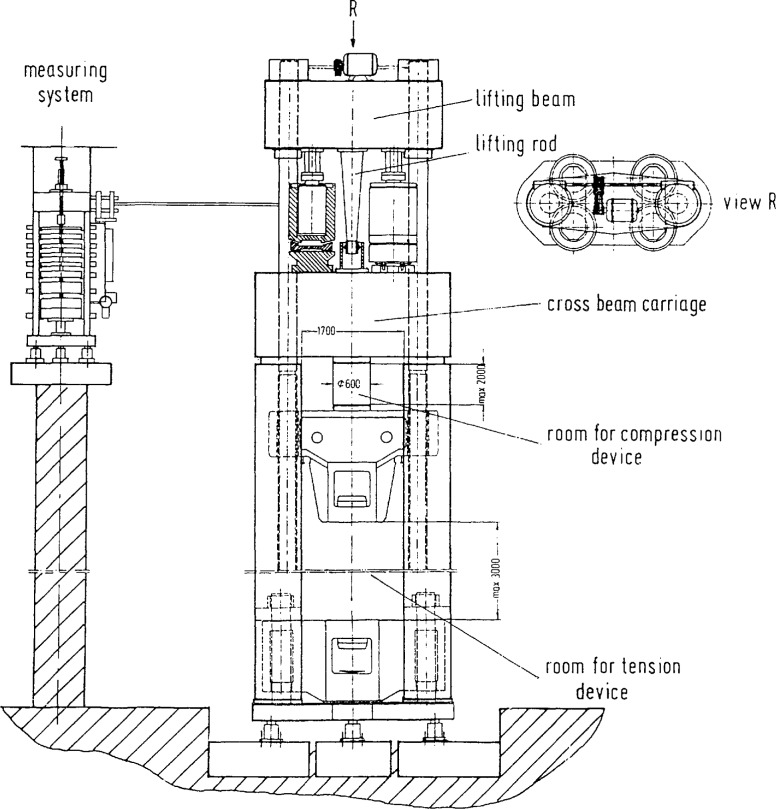
Schematic diagram of the PTB 15 MN hydraulie foree standard maehine.

A schematic diagram of the 1 MN deadweight machine is shown in Fig. 5. A schematic view of the 15 MN hydraulic force standard machine is shown in Fig. 6.

The overall uncertainty of the force realized in the hydraulic standard force machine has been estimated to be about 100 ppm [5]. The uncertainty of the 1 MN machine is comparable to that of the NIST deadweight machines.

## 3. Force Transfer Standards

A total of six force transducers were used in the intercomparison. Among these, five force transducers, having capacities of 100 kN, 200 kN, 500 kN, 1 MN, and 5 MN, have been in use at PTB over a substantial period of time. Accordingly, the behavior of these five force transducers is well known as is their long-term stability [1]. The history of the sixth transducer used, the 4.7 MN transducer, is not well documented. A complete description of each transducer can be found in Ref. [1].

To minimize the uncertainty associated with the indicating instrument a high-resolution indicator having a good stability was chosen. The resolution of the selected indicator is 1 ppm.

Prior to the start of the intercomparison, measurements were conducted at PTB, using a calibrating standard bridge to ascertain the reliability of the indicator when different main frequencies (50 and 60 Hz in Germany and USA, respectively) and different voltages (220 and 115 V in Germany and USA, respectively) are used. No significant differences were found.

## 4. Measurement Procedure

In developing the procedure utilized to perform the intercomparison, great care was given to minimize the effects of those parameters that are known to contribute to the measurement uncertainty. The following subsections describes these parameters and the ways in which their effects were minimized.

### 4.1 Time Interval

The difference between the output of a transducer at a load and its output when no load is applied represents the response of the transducer to that load. When a load is applied to a force transducer or when the force transducer is unloaded, there are initial mechanical, thermal and electrical responses in the various interconnected elements, followed by a delayed creep response or drift in the output of the transducer as the elements approach a new equilibrium condition. The process may be further complicated by local heating due to electrical-power dissipation by the strain-measuring bridge.

Although different force transducers exhibit different creep patterns [6], in general, the creep rate decreases greatly during the first few minutes following loading or unloading. To minimize the effect of creep, for each force transducer included in the intercomparison, the time required to achieve a stable response following loading and unloading was determined prior to the start of the intercomparison. In most instances it was found that a 3 min time delay between the initiation of the loading (or unloading) and the actual reading was adequate. When tests were conducted in the NIST 300 klbf and 1 Mlbf standard machines an additional 1.5 min was allowed to account for the longer loading and unloading times of these very-large deadweight machines.

In each instance, each set of measurements was duplicated once. In all cases a 3 min delay time was introduced between the completion of the initial set of measurements and the initiation of the duplicate set of measurements.

### 4.2 Machine-Transducer Interaction

Machine-transducer interactions can significantly influence measurement accuracy. Normal imperfections in the alignment of loading machines and force transducers can result in significant bending, shear, and twist components of deformation in the force transducer. To minimize the errors due to these nonaxial components of deformation, it is desirable to sample the response of the force transducer at several symmetrically distributed positions [7,8]. For this reason, the response of each force transducer was obtained at five positions relative to the axis of the machine (0°, 90°, 180°, 270°, 360°).

While at the 0° position and prior to the start of a measurement cycle, the force transducer was exercised by applying the maximum test load three times, returning to zero after each maximum load application. After a 3 min delay, with the force transducer still in the same position, two sets of measurements were obtained, each separated by a 3 min interval. Then, the force transducer was rotated by 90° and two new sets of measurements, each separated by a 3 min interval, were obtained, and so on. A special rotating mechanism, installed underneath the force transducer, allowed the transducer to be rotated rapidly through all the positions. During a measurement cycle (two sets of measurements at five positions) the force transducer was exercised only once at the beginning of the cycle at the 0° position.

### 4.3 Ambient Conditions

The measurements were carried out at (23 ± 0.5)°C, the usual laboratory conditions at NIST. Normally at PTB the laboratories are maintained at (20 ± 1) °C. However, a week prior to the initiation of the measurements, the temperature of the PTB laboratories was increased to (23 ± 1)°C. Both the force transducers and the indicator were kept at this temperature for a week prior to the initiation of measurements.

### 4.4 Force Steps

The forces realized in the PTB 15 MN standard machine were compared to the forces realized in the NIST 1 Mlbf deadweight machine. The forces realized in the PTB 1 MN deadweight machine were compared to those realized in the NIST 112 klbf, 300 klbf, and 1 Mlbf deadweight machines. The loads selected for machine intercomparison are listed in Tables 2, 3, and 4. Load selection was dictated by the constraints of the force standard machines intercompared, and the following criteria:

1. Limit the measurement range so that no measurements are made below 40% of the force transducer capacity. However, because of the limitation of the machines in some instances data were taken with loads as low as 20%.
2. Use only load sequences that can be applied monotonically;
3. For each force standard machine intercompared, select the same loads.

The following relationship was used to convert pound force to newtons:

1lbf=4.448222N.

## 5. Measurements Results

An effort was made to select for inclusion in the intercomparison similar loads at both PTB and NIST. However, because of machine limitations, the loads actually used, while similar, were significantly different as can be seen in Tables 2 through 4. For this reason, the NIST readings were normalized to correspond to the PTB applied force steps in accordance with the following equation:

$$\begin{array}{l} \begin{array}{l} \mathrm{NIST}\;\mathrm{normalized}\;\mathrm{indicator}\;\mathrm{reading}\\ \;=\frac{\mathrm{PTB}\;\mathrm{applied}\;\mathrm{force}\;\mathrm{kN}}{\mathrm{NIST}\;\mathrm{applied}\;\mathrm{force}\;\mathrm{kN}} \end{array}\\ \;\times \mathrm{NIST}\;\mathrm{indicator}\;\mathrm{reading}, \end{array}$$

where the NIST indicator reading is the net indicator reading obtained by subtracting the zero indicator reading from the reading at load.

### 5.1 Comparison of Forces in the Range of 50 to 500 kN

Three force transducers having nominal capacities of 100, 200, and 500 kN were used to intercom-pare, over a range of 50 to 500 kN, the forces realized in the NIST 112 klbf deadweight machine and the forces realized at PTB. The force steps selected for this intercomparison were 50, 100, 150, 200, 300, 400, and 500 kN.

The measurement variability in each series of measurements, at each force step and at each force transducer position, expressed as the relative data spread between runs, is given in Tables 5, 6, and 7 for the 100, 200, and 500 kN force transducers, respectively. The relative spread between runs was calculated by taking the difference between the first and second readings and dividing the result by the initial reading. Tables 5 through 7 show that, for each force step, the spread in the data, for all measurements performed at both NIST and PTB, is below 50 ppm.

The net mean force transducer outputs measured during the NIST measurements, the initial and final PTB measurements, and those obtained by averaging the initial and final PTB measurements are presented in Table 8. The values in Table 8 are in indicator units.

The relative differences between the average indicator readings at NIST and the corresponding average indicator readings at PTB are listed in Table 9 as a function of force transducer and force step. The values shown in Table 9 were obtained by taking the mean reading at NIST, subtracting from it the corresponding mean reading at PTB, and then dividing the result by the corresponding mean value of initial and final PTB readings. The values shown are rounded to the nearest ppm.

**Figure 7:**
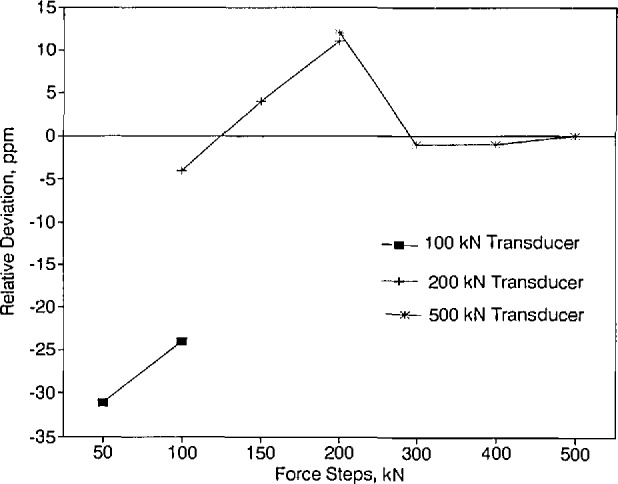
Deviations in the mean PTB data relative to the mean NIST data for the 50 to 500 kN range.

The average deviations in the mean PTB data relative to the mean NIST data for all force transducers and force steps examined (i.e., last column in Table 9) are given in Fig. 7. Figure 7 shows that the agreement between the data obtained at NIST and PTB is excellent. The deviations fall within a band ranging from −31 to 12 ppm.

### 5.2 Comparison of Forces in the Range of 90 kN to 1MN

Three force transducers having nominal capacities of 200 kN, 500 kN, and 1 MN were used to intercompare, over a range of 90 kN to 1 MN, the forces realized in the NIST 300 klbf deadweight machine and the forces realized at PTB. The force steps selected for this intercomparison were 90, 130, 180, 220, 310, 400, 410, 540, 670, 800, and 930 kN. When the program was planned originally, it was not anticipated that the 90, 130, and 180 kN force steps would be included in this subset of intercomparison. Accordingly, the first series of measurements performed at PTB did not include measurements for these force steps.

The measurement variability in each series of measurements, at each force step and each force transducer position, expressed as the relative data spread between runs, is given in Tables 10, 11, and 12 for the 200 kN, 500 kN, and 1 MN force transducers, respectively. The relative spread between runs was calculated by taking the difference between the first and second readings and dividing the result by the initial reading. Tables 10 through 12 show that, for each force step, the spread of the data is below 35 ppm for all measurements performed at both PTB and NIST.

The net mean force transducer outputs measured during the NIST measurements, the initial and final PTB measurements, and those obtained by averaging the initial and final PTB measurements are presented in Table 13. The values shown are in indicator units.

The relative differences between the average indicator readings at NIST and the corresponding readings at PTB are listed in Table 14 as a function of force transducer and force step. The values shown in Table 14 were obtained as described in Sec. 5.1 except that, for the 200 kN force transducer, the values shown were obtained by taking the mean reading at NIST, subtracting from it the corresponding mean final PTB reading, and dividing the result by the mean final PTB reading. All the values in Table 14 were rounded to the nearest ppm.

When evaluating the differences in the forces realized at PTB and NIST in the range between 220 and 400 kN, that is the forces determined using the 500 kN force transducer, only the relative differences between NIST and the final PTB data are meaningful because the initial set of measurements obtained at PTB were found to contain systematic errors. This is the reason why the decision was made to include the 200 kN force transducer in the set of force transducers measured in NIST 300 klbf deadweight machine.

**Figure 8:**
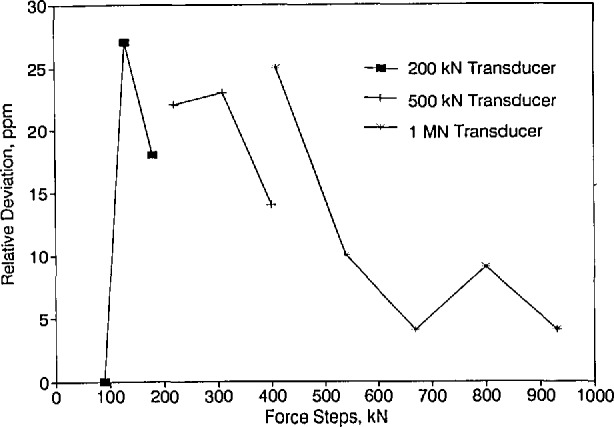
Deviations in the mean PTB data relative to the mean NIST data for the 90 to 1 MN range.

The average deviations in the mean PTB data relative to the mean NIST data for all force steps and all force transducers included in the 90 kN through the 1 MN range are shown in Fig. 8. For both the 200 and 500 kN force transducers, the values shown are based on the final PTB measurements only. The data shown for the 1 MN transducer are based on both the initial and final PTB measurements. Figure 8 shows that the agreement between the data obtained at NIST and PTB is very good. The deviations fall within a band from 0 to 30 ppm.

### 5.3 Comparison of Forces in the Range of 220 to 4500 kN

Three force transducers having nominal capacities of 1, 4.7, and 5 MN were used to intercompare, over a range of 220 to 4500 kN, the forces realized in the NIST 1 Mlbf machine and (a) the forces realized in the 1 MN PTB deadweight machine, and, (b) for forces above 1 MN, those achieved in the 15 MN PTB hydraulic multiplication standard machine. The force steps selected for this intercom-parison were 220, 440, 660, 880, 900, 1800, 2700, 3600, and 4500 kN.

The measurement variability in each series of measurements, at each force step and at each force transducer position, expressed as the relative data spread between runs, is given in Tables 15, 16, and 17 for the 1, 4.7, and 5 MN force transducers, respectively. The relative spread between runs was calculated in accordance with the procedure described in Sec. 5.1. Tables 15 through 17 show that for the 1 MN force transducer the largest spread, 57 ppm, was obtained at NIST at the 50 klbf force step. With the other two transducers, the largest spread was obtained at PTB with the maximum, 240 ppm, occurring with the 5 MN transducer at the 900 kN force step.

The net mean force transducer outputs measured at NIST and at PTB, and those obtained by averaging the initial and final PTB values are shown in Table 18. The values are in indicator units.

The relative differences between the average indicator readings at NIST and the corresponding average indicator readings at PTB are listed in Table 19 as function of force transducer and force step. The values shown were obtained in accordance with the procedure given in Sec. 5.1. All values shown in the table were rounded to the nearest ppm. Table 19 shows that overall there was good agreement between the data obtained at PTB and NIST. However, the response of the largest two transducers drifted somewhat during the course of the intercomparison. This drift was most significant with the 5 MN force transducer.

**Figure 9:**
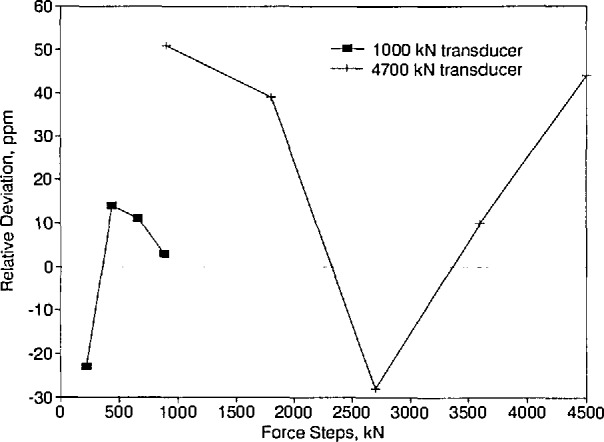
Deviations in the mean PTB data relative to the mean NIST data for the 220 kN to 4.5 MN range.

The average deviations in the mean PTB data relative to the mean NIST data for the 1 and 4.7 MN force transducers and force steps examined are shown in Fig. 9. Because of the drift observed in the 5 MN force transducer, the results obtained with this transducer were not included in the generation of Fig. 9. The average deviations fall within a band from −30 to 50 ppm.

## 6. Conclusions

Over a range of 50 to 4500 kN, the forces realized in the NIST deadweight machines compare favorably with those realized at PTB. Comparison of forces up to 900 kN indicate that the forces realized in the NIST deadweight machines and the forces realized in the PTB 1 MN deadweight machines agree within ±40 ppm. Peters et al. [7] have reported that theoretically the uncertainty in the forces realized by deadweight force machines is on the order of ±20 ppm. Accordingly, for the 50 to 900 kN force range, the agreement between the forces realized at NIST and PTB is close to what is theoretically achievable.

Comparison of forces in the range of 1 to 4.5 MN indicate that the forces realized in the PTB hydraulic force multiplication system compare favorably with the forces realized in the NIST deadweight machines. In this range, the agreement between the PTB data and the NIST data is within ±100 ppm, a remarkable agreement considering that the uncertainty of hydraulic force standard machine is inherently greater than that associated with deadweight machines.

## Figures and Tables

**Table 1:** Characteristics of the three largest NIST deadweight machines

Capacity: (nominal)			
kN	4448	1334	498
(klbf)	(1000)	(300)	(112)
Minimum load: (nominal)			
kN	222	44	13
(klbf)	(50)	(10)	(3)
Minimum increment: (nominal)			
kN	222	44	4.4
(klbf)	(50)	(10)	(1)
Time to capacity, s	345	292	274
Compression setup space:			
Vertical, m	1.98	1.65	1.02
Horizontal, m	0.86	0.91	0.71
Tension setup space:			
Vertical, m	4.45	2.49	2.16
Horizontal, m	1.17	0.91	0.71

**Table 2:** Load chosen to intercompare the PTB 1 MN and the NIST 112 klbf standard machines

Force transducer capacity, kN	Selected loads in PTB machine, kN	Selected loads in NIST machine	
		kN	(klbf)
100	50	48.9304	(11)
	100	97.8609	(22)
200	100	102.3091	(23)
	150	151.2395	(34)
	200	200.1700	(45)
500	200	200.1700	(45)
	300	298.0309	(67)
	400	400.3400	(90)
	500	498.2009	(112)

**Table 3:** Loads chosen to intercompare the PTB 1 MN and the NIST 300 klbf standard machines

Force transducer capacity, kN	Selected loads in PTB machine, kN	Selected loads in NIST machine	
		kN	(klbf)
200	90	88.9644	(20)
	130	133.4467	(30)
	180	177.9289	(40)
500	220	222.4111	(50)
	310	311.3755	(70)
	400	400.3400	(90)
1000	410	400.3400	(90)
	540	533.7866	(120)
	670	667.2333	(150)
	800	800.6800	(180)
	930	934.1266	(210)

**Table 4:** Load chosen to intercompare the PTB 1 MN, 15 MN and the NIST 1 Mlbf standard machines

Force transducer capacity, kN	Selected loads in PTB machine, kN	Selected loads in NIST machine	
		kN	(klbf)
1000	220	222.4111	(50)
	440	444.8222	(100)
	660	667.2333	(150)
	880	889.6444	(200)
4700	900	889.6444	(200)
	1800	1779.2888	(400)
and	2700	2668.9332	(600)
	3600	3558.5776	(800)
5000	4500	4448.2220	(1000)

**Table 5:** Relative data spread between runs for the 100 kN force transducer as a function of rotational position: 50 to 500 kN range

Institute	Applied force	Rotational position			
		0° ppm	90° ppm	180° ppm	270° ppm
PTB initial	50 kN	0	6	24	6
	100 kN	2	0	1	4
NIST	11 klbf	4	9	9	8
	22 klbf	4	17	11	6
PTB final	50 kN	14	29	31	5
	100 kN	4	6	2	9

**Table 6:** Relative data spread between runs for the 200 kN force transducer as a function of rotational position: 50 to 500 kN range

Institute	Applied force	Rotational position			
		0° ppm	90° ppm	180° ppm	270° ppm
PTB initial	100 kN	9	3	7	6
	150 kN	9	3	2	6
	200 kN	15	3	23	5
NIST	23 klbf	21	14	4	47
	34 klbf	8	4	1	5
	45 klbf	9	2	2	14
PTB final	100 kN	12	2	5	5
	150 kN	12	2	18	3
	200 kN	44	11	1	6

**Table 7:** Relative data spread between runs for the 500 kN force transducer as a function of rotational position: 50 to 500 kN range

Institute	Applied force	Rotational position			
		0° ppm	90° ppm	180° ppm	270° ppm
PTB initial	200 kN	1	4	1	10
	300 kN	8	2	3	7
	400 kN	10	2	1	3
	500 kN	9	1	0	1
NIST	45 klbf	13	1	9	21
	67 klbf	8	18	2	0
	90 klbf	5	7	4	4
	112 klbf	7	3	1	3
PTB final	200 kN	9	6	6	1
	300 kN	5	3	0	4
	400 kN	9	0	1	1
	500 kN	2	2	0	1

**Table 8:** Force transducer outputs measured at NIST and PTB: 50 to 500 kN range

Transducer kN	Force step kN	PTB initial	PTB final	PTB mean	NIST
100	50	1.069592	1.069593	1.069593	1.069559
	100	2.137819	2.137841	2.137830	2.137778
200	100	0.999438	0.999449	0.999444	0.999440
	150	1.499222	1.499257	1.499240	1.499246
	200	1.999049	1.999081	1.999065	1.999087
500	200	0.799180	0.799230	0.799205	0.799214
	300	1.198875	1.198944	1.198909	1.198908
	400	1.598594	1.598667	1.598630	1.598628
	500	1.998344	1.998420	1.998382	1.998382

**Table 9:** Relative differences between the NIST and PTB mean readings for the force transducer tested in the 112 klbf deadweight machine

Transducer kN	Force step kN	PTB initial ppm	PTB final ppm	PTB mean ppm
100	50	−31	−32	−31
	100	−19	−29	−24
200	100	2	−10	−4
	150	16	−8	4
	200	19	3	11
500	200	43	−19	12
	300	28	−30	−1
	400	21	−24	−1
	500	19	−19	0

**Table 10:** Relative data spread between runs for the 200 kN force transducer as a function of rotational position: 90 kN to 1 MN range

Institute	Applied force	Rotational position			
		0° ppm	90° ppm	180° ppm	270° ppm
PTB final	90 kN	3	11	6	23
	130 kN	21	8	3	8
	180 kN	1	13	3	2
NIST	20 klbf	0	1	3	3
	30 klbf	6	4	1	4
	40 klbf	11	4	2	1

**Table 11:** Relative data spread between runs for the 500 kN force transducer as a function of rotational position: 90 kN to 1 MN range

Institute	Applied force	Rotational position			
		0° ppm	90° ppm	180° ppm	270° ppm
PTB initial	220 kN	5	5	1	16
	310 kN	9	2	10	13
	400 kN	1	1	13	9
NIST	50 klbf	0	4	4	4
	70 klbf	2	3	5	5
	90 klbf	5	10	2	4
PTB final	220 kN	2	1	3	6
	310 kN	2	6	2	2
	400 kN	6	1	3	1

**Table 12:** Relative data spread between runs for the 1 MN force transducer as a function of rotational position: 90 kN to 1 MN range

Institute	Applied force	Rotational position			
		0° ppm	90° ppm	180° ppm	270° ppm
PTB initial	410 kN	2	17	0	0
	540 kN	3	25	25	31
	670 kN	3	9	2	10
	800 kN	1	10	2	4
	930 kN	3	2	2	6
NIST	90 klbf	5	27	5	20
	120 klbf	0	7	2	2
	150 klbf	4	0	3	2
	180 klbf	5	1	1	4
	210 klbf	2	4	4	5
PTB final	410 kN	2	0	1	6
	540 kN	12	8	1	4
	670 kN	2	8	7	4
	800 kN	10	4	2	1
	930 kN	13	8	4	2

**Table 13:** Force transducer outputs measured at NIST and PTB: 90 kN to 1 MN range

Transducer kN	Force step kN	PTB initial	PTB final	PTB mean	NIST
200^a^	90		0.899500		0.899500
	130		1.299331		1.299366
	180		1.799171		1.799204
500^b^	220	0.879081	0.879165	0.879123	0.879184
	310	1.238808	1.238898	1.238853	1.238927
	400	1.598538	1.598644	1.598591	1.598666
1000	410	0.835596	0.835609	0.835602	0.835623
	540	1.100592	1.100621	1.100607	1.100618
	670	1.365630	1.365669	1.365649	1.365654
	800	1.630647	1.630694	1.630670	1.630684
	930	1.895624	1.895674	1.895649	1.895656

a No initial PTB measurements were taken with this transducer.

b Initial PTB measurements contained systematic errors and should not be used to intercompare the forces achieved in the NIST 300 klbf deadweight machine with those achieved at PTB.

**Table 14:** Relative differences between NIST and PTB readings for the force transducers tested in the 300 klbf deadweight machine

Transducer kN	Force step kN	PTB initial ppm	PTB final ppm	PTB mean ppm
200^a^	90		0	
	130		27	
	180		18	
500^b^	220	117	22	69
	310	96	23	60
	400	80	14	47
1000	410	32	17	25
	540	24	−3	10
	670	18	−11	4
	800	23	−6	9
	930	17	−9	4

a No initial PTB measurements were taken with this transducer.

b Initial PTB measurements contained systematic errors and should not be used to intercompare the forces achieved in the NIST 300 klbf deadweight machine with those achieved at PTB.

**Table 15:** Relative data spread between runs for the 1 MN force transducer as a function of rotational position: 220 to 880 kN range

Institute	Applied force	Rotational position			
		0° ppm	90° ppm	180° ppm	270° ppm
PTB initial	220 kN	4	2	13	7
	440 kN	3	11	10	8
	660 kN	16	5	8	5
	880 kN	3	3	2	1
NIST	50 klbf	31	51	53	57
	100 klbf	17	19	19	13
	150 klbf	4	1	9	25
	200 klbf	4	0	14	9
PTB final	220 kN	0	4	9	27
	440 kN	2	4	4	7
	660 kN	1	11	9	1
	880 kN	5	6	7	0

**Table 16:** Relative data spread between runs for the 4.7 MN force transducer as a function of rotational position

Institute	Applied force	Rotational position			
		0° ppm	90° ppm	180° ppm	270° ppm
PTB initial	900 kN	95	54	27	27
	1800 kN	48	34	27	14
	2700 kN	64	130	140	27
	3600 kN	37	58	51	20
	4500 kN	35	38	44	14
NIST	200 klbf	25	33	39	33
	400 klbf	17	29	25	18
	600 klbf	34	2	6	6
	800 klbf	28	6	2	2
	1000 klbf	32	8	0	5
PTB final	900 kN	27	110	68	95
	1800 kN	0	14	48	0
	2700 kN	0	59	23	32
	3600 kN	7	37	10	24
	4500 kN	3	16	22	38

**Table 17:** Relative data spread between runs for the 5 MN force transducer as a function of rotational position

Institute	Applied force	Rotational position			
		0° ppm	90° ppm	180° ppm	270° ppm
PTB initial	900 kN	81	90	18	27
	1800 kN	4	27	13	27
	2700 kN	6	42	15	18
	3600 kN	20	2	20	16
	4500 kN	14	29	7	13
NIST	200 klbf	11	38	42	82
	400 klbf	33	25	25	25
	600 klbf	1	51	1	16
	800 klbf	0	37	28	1
	1000 klbf	12	29	29	14
PTB final	900 kN	110	54	170	240
	1800 kN	0	130	9	130
	2700 kN	99	27	110	99
	3600 kN	36	72	83	130
	4500 kN	23	23	63	22

**Table 18:** Force transducer outputs measured at NIST and PTB: 220 to 4500 kN range

Transducer MN	Force step kN	PTB initial	PTB final	PTB mean	NIST
1	220	0.448320	0.448304	0.448312	0.448302
	440	0.896749	0.896739	0.896744	0.896757
	660	1.345272	1.345269	1.345270	1.345285
	880	1.793741	1.793745	1.793743	1.793748
4.7	900	0.557929	0.557866	0.557898	0.557926
	1800	1.115960	1.115896	1.115928	1.115971
	2700	1.673944	1.674003	1.673974	1.673927
	3600	2.231629	2.231759	2.231694	2.231717
	4500	2.789248	2.789319	2.789284	2.789406
5	900	0.367344	0.367306	0.367325	0.367351
	1800	0.734166	0.734051	0.734108	0.734212
	2700	1.101155	1.100933	1.101044	1.101248
	3600	1.468967	1.468721	1.468844	1.469005
	4500	1.837706	1.837474	1.837590	1.837671

**Table 19:** Relative differences between the NIST and PTB readings for the force transducers tested in the 1 Mlbf deadweight machine

Transducer MN	Force step kN	PTB initial ppm	PTB final ppm	PTB mean ppm
1	220	−40	−5	−23
	440	9	20	14
	660	10	12	11
	880	4	2	3
4.7	900	−5	110	51
	1800	10	68	39
	2700	−11	−46	−28
	3600	40	−19	10
	4500	57	31	44
5	900	17	120	69
	1800	63	220	140
	2700	84	290	190
	3600	26	190	110
	4500	−19	110	44
